# Global burden of moyamoya syndrome in pediatric sickle cell disease and implications for the neurosurgical workforce: a systematic review

**DOI:** 10.1007/s00381-026-07475-7

**Published:** 2026-09-22

**Authors:** Joseline Haizel-Cobbina, Anne Heukwa-Tefoung, Soumya Pahari, Ikeoluwa Lagunju, Lori C. Jordan, Philip Aldana, Michael C. Dewan

**Affiliations:** 1 Department of Neurosurgery, Vanderbilt University Medical Center, Nashville, TN USA; 2 Vanderbilt Institute for Global Health, Vanderbilt University Medical Center, Nashville, TN USA; 3 Oakland University William Beaumont School of Medicine, Rochester, MI USA; 4 Department of Surgery, Neurosurgical Unit, Nepalese Army Institute of Health Sciences, Kathmandu, Nepal; 5 Paediatric Neurology Unit, Department of Paediatrics, College of Medicine, University of Ibadan, Ibadan, Oyo Nigeria; 6 Division of Pediatric Neurology, Department of Pediatrics, Vanderbilt University Medical Center, Nashville, TN USA; 7 Division of Pediatric Neurosurgery, University of Florida, Jacksonville, FL USA; 8 Division of Pediatric Neurological Surgery, Department of Neurological Surgery, Doctors Office Tower, Vanderbilt Children’s Hospital, 2200 Children’s Way, 9226, Nashville, TN 37232-9557 USA

**Keywords:** Sickle cell disease, Moyamoya syndrome, Pediatric, Surgical revascularization

## Abstract

**Objective:**

The aims of the study were twofold: 1) quantify the global burden of pSCD|MMS, and 2) estimate the number of children who warrant specialist evaluation for possible surgical revascularization.

**Methods:**

A systematic review was conducted following PRISMA guidelines. Clinical data extracted included number of patients with pSCD, pSCD-MMS, and those who underwent surgical revascularization. Using weighted proportions and 2021 Institute for Health Metrics and Evaluation (IHME) global estimates for pSCD, for each WHO region we estimated the burden of pSCD|MMS, and the volume of pSCD|MMS patients who underwent surgical revascularization.

**Results:**

Of the 6,845 studies identified, 14 were included. Nine (64%) studies were from AMR, 2 (14%) studies from EMR, 1 study (7%) from EUR, 1 study (7%) from AFR, and 1 (7%) multicenter study from AMR and EUR. Of the 2045 pSCD patients between the ages of 1–21 years identified, 1.5% were diagnosed with pSCD|MMS. Of 279 pSCD|MMS patients, 49.8% underwent surgical revascularization. The projected number of pSCD|MMS cases worldwide was 36,587, equating to 18,219 potentially benefiting from surgical revascularization. Across WHO regions, the African region harbors the largest number of projected pSCD|MMS cases (32,189) including 16,030 candidates for surgical consideration. Projections should be interpreted cautiously given the limited number and geographic distribution of the available studies.

**Conclusion:**

The vast majority of pSCD and pSCD|MMS burden resides in Africa, wherein SCD-related infrastructure is often limited. Sufficient capacity building for diagnostic accuracy and screening coupled with maximal medical therapy for pSCD, and robust neurosurgical infrastructure is required to improve longitudinal SCD care, mitigate neurologic complications and enhance functional outcomes. Derived from a limited body of published literature, these estimates should be considered exploratory.

**Supplementary Information:**

The online version contains supplementary material available at https://doi.org/10.1007/s00381-026-07475-7.

## Introduction

Cerebrovascular events, including ischemic and hemorrhagic stroke, occur in about 11% of pediatric SCD patients (pSCD) by age 20 [1]. Strokes may arise from small-vessel disease, large-vessel disease, and impairment of cerebral autoregulation [2]. Moyamoya syndrome (MMS) is a large-vessel disease complication in SCD characterized by progressive occlusion of the supraclinoid internal carotid arteries and their major branches with development of collateral vessels at the base of the brain [3]. Pediatric SCD patients with MMS (pSCD|MMS) have a two-fold risk of experiencing a recurrent stroke and transient ischemic attacks (TIA) compared to pSCD without MMS [4, 5]. They also have relatively worse neuropsychological outcomes than SCD children without MMS [5, 6].

Two landmark trials in the United States, the Stroke Prevention Trial in Sickle Cell Anemia (STOP) and the TCD with Transfusions Changing to Hydroxyurea trial (TWiTCH), demonstrated that chronic blood transfusions[7] and subsequent transition to hydroxyurea therapy were effective in primary stroke prevention in pSCD with abnormal Transcranial Doppler (TCD) velocities (> 200 cm/second), notably in TWiTCH, children with significant cerebral vasculopathy such as pSCD|MMS were excluded from transition to hydroxyurea and continued on lifelong transfusion therapy [8]. Both an observational study and a phase III clinical trial in Nigeria showed that hydroxyurea is beneficial for primary stroke prevention in pSCD but did not specifically focus on children with pSCD|MMS [9, 10], The efficacy of medical management in pSCD|MMS remains limited with roughly 60–90% of patients still experiencing a stroke despite appropriate medical therapy [11–13]. While large multi-center studies are unavailable, smaller surgical case series suggest that cerebral revascularization in combination with conservative management may be effective in preventing stroke recurrence in pSCD|MMS [12–14].

To support and justify global capacity building efforts to improve access to care and outcomes for pSCD|MMS, we sought to quantify the global burden of pSCD|MMS, and to estimate the number of children who warrant specialist evaluation for surgical revascularization.

## Methods

### Database searches

A systematic review and meta-analysis was conducted following the Preferred Reporting Items for Systematic Review and Meta-Analyses (PRISMA) utilizing MeSH terms for “sickle cell disease”, “Moyamoya syndrome”, and “surgical revascularization”. A comprehensive literature search was conducted in December 2023 [15]. Databases queried include PubMed (National Library of Medicine), Cumulative Index of Nursing and Allied Health Literature (CINAHL), Embase (Elsevier), Web of Science, Cochrane CENTRAL Register of Controlled Trials and Database of Systematic Reviews, and WHO Global Index Medicus. There were no restrictions on language, year of publication or study design applied to the search. The Supplemental File provides a comprehensive list of search terms.

### Article screening

Duplicates were removed via an automated duplication detection feature in Rayyan Systems Inc. followed by manual resolution of duplicates [16]. Title and abstract screening followed by full text article screening were conducted by S.P and A.H.T At both stages of screening, each article was reviewed by a minimum of two authors to ensure a comprehensive review. Conflicts were resolved by J.H.C and M.C.D. Inclusion criteria were 1) articles reporting on pediatric populations with SCD (age ≤ 21 years), 2) articles reporting on pediatric patients with SCD with MMS, 3) articles reporting on pSCD|MMS patients undergoing revascularization surgery. Exclusion criteria were: 1) articles reporting on SCD|MMS in the adult population only, 2) articles reporting on pediatric patients with moyamoya disease or non-SCD causes of MMS, 3) articles reporting on only a preselected cohort (e.g. only pSCD|MMS cohorts or only cases which underwent surgical intervention), and 4) letters to the editor, literature reviews, meta-analyses, conference abstracts, or narrative reports. No article was excluded on the basis of language.

### Data abstraction

The final set of articles selected after the screening were reviewed for relevant data by three reviewers (J.H.C, A.H.T, and S.P). Bibliometric data on authors, year of publication, study design, and country of publication were extracted. World Health Organization (WHO) region and World Bank income group of the country of origin for each article were reported based on the WHO designations: Region of the Americas (AMR), European Region (EUR), African Region (AFR), Western Pacific Region(WPR), South-East Asia Region (SEAR) and Eastern Mediterranean Region (EMR); and World Bank income classification: low income countries (LICs), lower and upper middle income countries (lower and upper MICs) and high income countries (HICs) [17, 18]. Clinical data extracted included: number of patients with pSCD, number of patients with pSCD|MMS, and number of patients who underwent surgical revascularization. Data on the burden of pSCD patients aged 0–14 years were obtained for each WHO region obtained from the Institute of Health Metrics and Evaluation (IHME) 2021 global burden of disease estimates [19]. Quality and risk of bias in the included studies were assessed using the GRADE framework and ROBINS-I tool [20, 21]. The study protocol was registered in the PROSPERO database (Registration number: CRD420261328516.)

### Data reporting and quantitative analysis

Descriptive data reported across the studies identified was used to estimate the weighted proportions of 1) pSCD|MMS and 2) pSCD|MMS patients who underwent surgical revascularization as outlined below:

1. To estimate the proportion of pSCD|MMS cases:
  a The weight for each study (*sw*) = study sample size (*ss*)/sum of sample sizes of all included studies (*S*)
  b The proportion of pSCD|MMS cases for each study = number of pSCD|MMS/total number of pSCD
  c The weighted average proportion (*WP)* of pSCD|MMS = sum (*proportion of* pSCD|MMS x *sw*) across all studies included.
2. To estimate the proportion of pSCD|MMS who underwent surgical revascularization:
  a The weight for each study which reported surgical data (*sw*) = study sample size (*ss*)/sum of sample sizes of all included studies (*S*)
  b The proportion of pSCD|MMS who underwent surgical intervention for each study = number of pSCD|MMS who underwent surgical revascularization/total number of pSCD|MMS
  c The *WP* of pSCD|MMS who underwent surgical revascularization = sum (proportion of pSCD|MMS who underwent surgical revascularization x *sw*) across all studies included.

Using the 2021 IHME estimates of the burden of pSCD for each WHO region and the weighted proportions computed above, we estimated the burden of pSCD|MMS and the proportion of cases who warrant specialist evaluation for surgical revascularization in each WHO region as follows:

1. pSCD|MMS _WHO region_ = pSCD Burden _WHO region_ X *WP* (pSCD|MMS)
2. pSCD|MMS requiring surgical evaluation _WHO region_ = pSCD|MMS _WHO region_ X *WP* (pSCD|MMS requiring surgical evaluation)

## Results

**Fig. 1:**
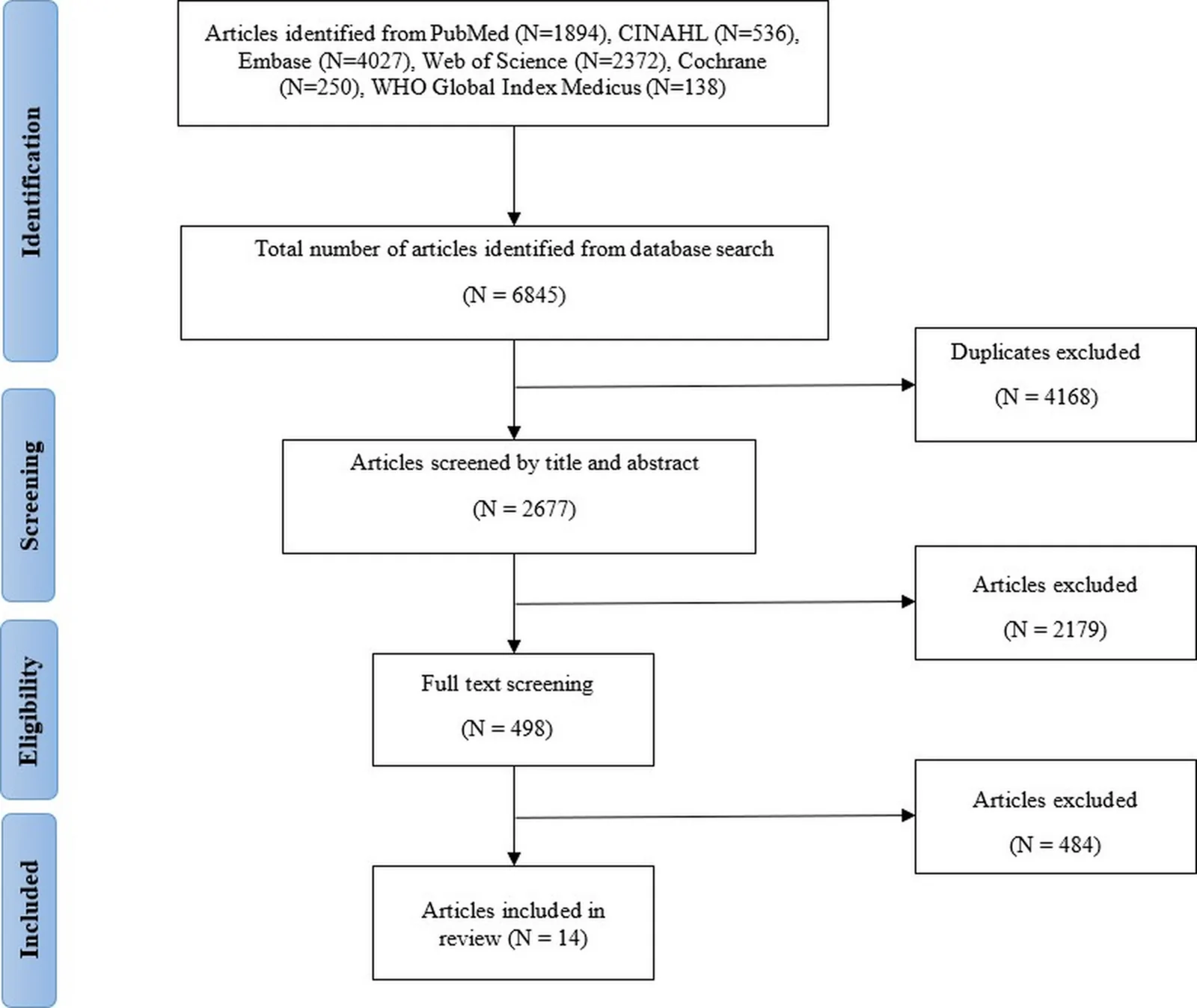
PRISMA flow diagram for the literature search

The literature search yielded 6,845 studies out of which 2,677 duplicates were removed. Following title and abstract screening 498 articles were selected for full text review. Fourteen unique articles were included in the study after the full text review (Fig. 1). By WHO region, 9 (64%) studies were from AMR, 2 (14%) studies from EMR, 1 study (7%) from EUR, 1 study (7%) from AFR, and 1 (7%) multicenter study from AMR and EUR. With the exception of 1 study from a lower MIC region, all other studies were from HICs. The majority of the studies included were retrospective cohort studies (64%, n = 9). Other study types included cross sectional studies (2), prospective cohort study (1), retrospective case series (1), and case–control study (1). Using the GRADE framework for assessments, 14% of studies included in the review very low quality, 50% of studies had low quality, and 36% of studies had moderate quality. Based on the ROBINS-I tool, 14% of articles assessed had a low risk of bias, 75% had a moderate risk of bias, and 5% had a serious risk of bias. Using the GRADE framework for assessments [20], 14% of studies included in the review had very low quality, 50% of studies had low quality, and 36% of studies had moderate quality. Based on the ROBINS-I tool [21], 86% of articles assessed had a moderate risk of bias and 14% had a serious risk of bias.

Five articles from 4 countries in AFR, EUR, EMR, and AMR reported data on pSCD-MMS (Table 1). Out of 2045 patients with pSCD of hemoglobin SS and SC genotype between the ages of 1–21 years, 30 of them were diagnosed with MMS on MR angiography (MRA) with a weighted proportion of 1.5%. All 30 patients diagnosed with MMS had hemoglobin SS genotype. Ten articles from 4 countries in EMR, EUR, and AMR reported data on surgical revascularization (Table 2). Out of 279 pSCD|MMS patients between the ages of 1–21 years, 139 (49.8%) underwent surgical revascularization.

**Table 1:** Overview of articles reporting data on pSSD with MMS

Author and year	Country	Income	WHO region	Study design	Single/multi-institution	Age	# pSCD cases	# pSCD|MMS	Grade	Risk of bias
Moser et al., 1996^42^	USA	HIC	AMR	Cross sectional study	Multi	6 −14	312	3	Low	Moderate
Hines et al., 2011^43^	USA	HIC	AMR	Retrospective cohort study	Single	5—21	73	1	Moderate	Moderate
Bader-Meunier et al., 2008^36^	France	HIC	EUR	Retrospective cohort study	Single	2—14	1075	9	Low	Moderate
Zayed et al., 2023^44^	Saudi Arabia	HIC	EMR	Retrospective cohort study	Single	1—14	385	16	Low	Moderate
Kija et al., 2019^45^	Tanzania	Lower MIC	AFR	Cross sectional study	Single	6—18	200	1	Moderate	Moderate

WHO: World Health Organization; AMR: America Region; HIC: High income country; MIC: Middle income country; AFR: Africa Region; EMR: Eastern Mediterranean Region; EUR: European Region; pSCD|MMS: pediatric sickle cell disease with moyamoya syndrome

**Table 2:** Overview of articles reporting data on surgical intervention in pSSD with MMS

Author and year	Country	Income	WHO region	Study design	Single/multi-institution	Age	# pSCD|MMS	**# **Revascularization	Grade	Risk of bias
Hulbert et al., 2011^34^	USA, UK	HIC	AMR, EUR	Prospective cohort study	Multi	2—16	9	4	Moderate	Moderate
Aldana et al., 2023^14^	USA	HIC	AMR	Retrospective cohort study	Multi	3–8	137	78	Moderate	Moderate
Gatti et al., 2021^46^	USA	HIC	AMR	Retrospective cohort study	Single	7 ± 4	18	9	Low	Moderate
Yang et al., 2017^13^	USA	HIC	AMR	Retrospective cohort study	Single	2—16	15	8	Low	Moderate
Winstead et al., 2017^47^	USA	HIC	AMR	Retrospective cohort study	Single	5–19	11	7	Very Low	Serious
Griessenauer et al., 2015^12^	USA	HIC	AMR	Retrospective cohort study	Single	5—21	22	14	Low	Moderate
Kaseka et al., 2021^48^	Canada	HIC	AMR	Case–control study	Single	6—12	21	3	Moderate	Moderate
Hall et al., 2016^49^	USA	HIC	AMR	Retrospective cohort study	Multi	5—10	26	12	Low	Moderate
Saeed, 2016^50^	Saudi Arabia	HIC	EMR	Retrospective case series	Single	1—11	4	3	Very Low	Serious
Zayed et al., 2023^44^	Saudi Arabia	HIC	EMR	Retrospective cohort study	Single	1—14	16	1	Low	Moderate

WHO: World Health Organization; AMR: America Region; HIC: High income country; EMR: Eastern Mediterranean Region; EUR: European Region; pSCD|MMS: pediatric sickle cell disease with moyamoya syndrome

Based on the 2021 IHME report on the burden of pSCD, there were 2,439,140 pSCD cases worldwide with AFR having the highest burden of cases (2,145,964) followed by EMR with 220,406 cases. SEAR had the least burden of pSCD with 65 cases reported. Using the estimated weighted proportion of pSCD|MMS and pSCD|MMS requiring evaluation for surgical revascularization above, the projected number of pSCD|MMS cases worldwide was 36,587 with 18,219 requiring evaluation for surgical revascularization. Across WHO Regions, countries in AFR had the largest number of projected pSCD|MMS cases at 32,189 projected cases with 16,030 requiring surgical evaluation (Table 3).

**Table 3:** Estimated annual case burden of pSCD|MMS by WHO region

WHO region	pSCD cases (0–14 years)	Number of projected pSCD|MMS cases	Projected number of pSCD|MMS surgical cases
AFR	2,145,964	32,189	16,030
AMR	52,220	783	390
EMR	220,406	3306	1646
EUR	11,904	179	89
SEAR	65	0.97	0.48
WPR	8581	129	64
Worldwide	2,439,140	36,587	18,219

WHO: World Health Organization; AMR: America Region; AFR: Africa Region; EMR: Eastern Mediterranean Region; EUR: European Region; SEAR: Southeast Asia Region; WPR: Western Pacific Region; pSCD: pediatric sickle cell disease; pSCD|MMS: pediatric sickle cell disease with moyamoya syndrome

## Discussion

To our knowledge this is the first study to define the global burden of pSCD|MMS and proportion of patients who may benefit from surgical revascularization globally. Summarily, based upon the available published literature, approximately 1.5% of all pSCD cases are estimated to be diagnosed with MMS out of which nearly half could benefit from surgical revascularization. Projecting the global burden based on these estimates, there are more than 36,000 pSCD|MMS projected cases worldwide, half of whom should be evaluated for surgery. The majority of pSCD|MMS were found in AFR, which bears the greatest burden of SCD in both the pediatric and adult populations [22].

The distribution of pSCD varies across regions with an expectedly high prevalence in regions where malaria is endemic including sub-Saharan Africa, the Mediterranean, the Middle East, and India [23]. However, with increased global migration, pSCD prevalence is changing in various regions with an increasing disease burden reported in the Americas and Europe [19]. The true prevalence of pSCD|MMS remains underestimated in many parts of the world including AFR due to limited diagnostic tools and lack of surveillance [24]. Accordingly, these estimates more closely reflect diagnosed MMS rather than true biological prevalence. The pooled estimate of pSCD|MMS in our study is lower than what was reported in a previous single center study evaluating abnormal brain imaging findings in the pSCD cohort (1.5% vs. 55%) [25]. Several HIC and LMIC studies have reported a higher pSCD|MMS prevalence (43%—96%) among pSCD patients diagnosed with a stroke [4, 26]. This may suggest a poor implementation of TCD screening for stratifying stroke risk even in well-resourced countries with established national screening protocols [27, 28].

The importance of TCD screening and medical management to prevent and/or reduce the incidence of neurologic complications in pSCD cannot be overemphasized and should be well established before even considering surgical capacity building specifically for revascularization. An African study with the largest pSCD|MMS cohort in the region showed that neurologic complications including first stroke occurrence was more common in preschool and school-aged children [26]. Current evidence-based recommendations by the National Heart, Lung, and Blood Institute (NHLBI) is to start TCD screening and medical intervention at 2 years of age to help with early identification and intervention for children with pSCD at risk of neurologic complications [29, 30]. Preliminary results from the Stroke Prevention in Nigeria (SPIN) feasibility trial using moderate fixed-dose hydroxyurea therapy (~ 20 mg/kg/day) demonstrated that hydroxyurea therapy is safe, feasible, and effective at reducing elevated TCD velocities which indicates a lower risk of stroke [31]. As part of capacity building efforts for primary stroke prevention effort in Nigeria, a task shifting TCD training program was implemented due to limited availability of health care providers with the needed expertise to perform TCD [32, 33]. This initiative, which trained nurses and non-specialist medical officers to conduct TCD screening provides a viable option for building capacity prevent and/or reduce the burden of neurologic complications in pSCD in SSA and other resource-limited settings [32, 33].

While medical intervention significantly reduces the risk of stroke in pSCD, it may not be as effective in preventing stroke in patients who have pSCD|MMS. Previous studies have shown that despite optimal chronic transfusions and hydroxyurea therapy, many patients with severe pSCD|MMS still experience ischemic complications [34–36]. Based on the pooled results, almost 50% of pSCD|MMS patients needed surgical revascularization in a setting where they had access to optimal medical therapy. In a comparative study by Aldana *et al*^*14*^, surgical revascularization was shown to be associated with a 3.7-fold reduction in the odds of stroke in pSCD|MMS compared to patients who received medical intervention alone [14]. There are currently no standard clinical guidelines to determine the indications for surgical revascularization. However, a recent Delphi panel recommends surgery when there is at least 50% arterial stenosis matching the distribution of an ischemic stroke or transient ischemic attack (TIA) together with the presence of moyamoya collateral vessels [37].

The success of surgical revascularization in preventing stroke recurrence and optimizing functional outcomes in pSCD|MMS requires a skilled neurovascular team, careful patient selection, and diligent anesthetic and intensivist care. This poses a treatment challenge in resource-limited settings, especially AFR wherein there is a disproportionate burden of cases alongside lean and inadequate infrastructure for cerebral revascularization surgery [38–40]. Despite this challenge, the available evidence supports the notion that access to revascularization surgery represents a crucial – even if aspirational – component of stroke prevention in pSCD|MMS [14, 37, 41]. While this report offers an estimate of disease burden, formal workforce planning will require additional region-specific information regarding existing personnel, infrastructure, referral systems, operative capacity, and training resources, all of which deserve their own interrogation and estimation.

Beyond the in-hospital workup and management of MMS, the importance of community awareness and early recognition deserves emphasis. Indeed, advanced screening pathways or the expansion of revascularization services would be grossly underutilized in the absence of the awareness of neurological complications among patients, families, and frontline healthcare providers. Community healthcare providers are typically well poised to provide culturally appropriate education in the local language and engage with families in such a way that local beliefs and traditions are respected. Thus, strenghthening community-based education is a logical early step aimed at reducing delays in diagnosis and improving access to specialized care.

This study has several limitations that could affect the accuracy of our estimations. Estimates were derived largely from small sized HIC studies due to the paucity of publications and primary data from low resource settings especially AFR and EMR regions where pSCD is endemic. While beyond the scope of this manuscript, further studies incorporating nontraditional sources such as regional registries and Ministry of Health datasets may increase the accuracy of global SCD|MMS estimates. The limited data also made it difficult to estimate the regional differences in pSCD|MMS and the surgical burden. The surgical series used to estimate the proportion of pSCD|MMS requiring surgery may suffer from referral bias resulting in overestimation of this proportion. Larger studies are needed, including multi-institutional and multi-national studies to estimate the true prevalence of pSCD|MMS and the associated neurologic complications and long-term outcomes. This review intentionally did not delve into the varied manners of revascularization (including direct anastomosis and the many forms of indirect revascularization) though the authors believe there may be value in a tiered approach to revascularization guided by the existing capacity (eg. operative microscope, intraoperative neuromonitoring) at any given facility. Despite these limitations, this study sheds light on the global burden of pSCD|MMS to catalyze advocacy efforts aimed at improving access to care for this patient population.

## Conclusion

A small but significant proportion of sickle cell patients develop moyamoya syndrome, nearly half of whom could benefit from revascularization surgery. The vast majority of the sickle cell disease-related moyamoya syndrome burden resides in Africa, wherein SCD-related infrastructure is often limited. Improvement in longitudinal SCD care including diagnostic accuracy and screening, optimal medical therapy, and strengthening neurosurgical capacity are needed to mitigate neurologic complications and improve functional outcomes.

## Supplementary Information

Below is the link to the electronic supplementary material.

ESM 1 (DOCX 35.7 KB)

## Data Availability

No datasets were generated or analysed during the current study.
