# Supplementary material for: Global burden of moyamoya syndrome in pediatric sickle cell disease and implications for the neurosurgical workforce: a systematic review
Source: Childs Nerv Syst. 2026 Sep 22;42(1):370. doi: 10.1007/s00381-026-07475-7 (PMC13597574; doi:10.1007/s00381-026-07475-7)
Supplement: Supplementary file 1 [file 381_2026_7475_MOESM1_ESM.docx]

Table 1: List of MeSH terms

| **PubMed (NCBI):**  **Results: 1,894**  **Limits/Filters: Children** | ("Anemia, Sickle Cell"[Mesh] OR drepanocyte[tiab] OR drepanocytemia[tiab] OR drepanocytes[tiab] OR drepanocytic anaemia[tiab] OR drepanocytic anemia[tiab] OR drepanocytosis[tiab] OR haemoglobin c[tiab] OR haemoglobin d[tiab] OR haemoglobin s[tiab] OR haemoglobin sc[tiab] OR haemoglobin sd[tiab] OR haemoglobin ss[tiab] OR Hb C[tiab] OR Hb D[tiab] OR Hb S[tiab] OR Hb SC[tiab] OR Hb SD[tiab] OR Hb SS[tiab] OR HbC[tiab] OR HbD[tiab] OR HbS[tiab] OR HbSC[tiab] OR HbSD[tiab] OR HbSS[tiab] OR hemoglobin c[tiab] OR hemoglobin d[tiab] OR hemoglobin s[tiab] OR hemoglobin sc[tiab] OR hemoglobin sd[tiab] OR hemoglobin ss[tiab] OR hemoglobinopathy c[tiab] OR hemoglobinopathy d[tiab] OR hemoglobinopathy s[tiab] OR hemoglobinopathy sc[tiab] OR hemoglobinopathy sd[tiab] OR hemoglobinopathy ss[tiab] OR meniscocyte[tiab] OR meniscocytes[tiab] OR meniscocytosis[tiab] OR sickle anaemia[tiab] OR sickle anemia[tiab] OR sickle cell[tiab] OR sickle cells[tiab] OR sickle erythrocyte[tiab] OR sickle erythrocytes[tiab] OR sickled[tiab] OR sickler[tiab] OR sickling trait[tiab]) AND ("Brain Infarction"[Mesh] OR "Brain Ischemia"[Mesh] OR "Intracranial Embolism and Thrombosis"[Mesh] OR "Intracranial Hemorrhages"[Mesh] OR "Moyamoya Disease"[Mesh] OR "Stroke"[Mesh] OR "Vasospasm, Intracranial"[Mesh] OR acute cerebrovascular lesion[tiab] OR acute cerebrovascular lesions[tiab] OR acute focal cerebral vasculopath*[tiab] OR apoplectic[tiab] OR apoplex*[tiab] OR brain event[tiab] OR brain events[tiab] OR cardio-vascular event[tiab] OR cardio-vascular events[tiab] OR cardiovascular event[tiab] OR cardiovascular events[tiab] OR corpus callosum hemorrhag*[tiab] OR cortical infarct*[tiab] OR CVA[tiab] OR CVAs[tiab] OR encephalorrhagia[tiab] OR hematencephalon[tiab] OR insultus cerebralis[tiab] OR intracortical hemorrhag*[tiab] OR ischaemic attack[tiab] OR ischaemic attacks[tiab] OR ischaemic encephalopathies[tiab] OR ischaemic encephalopathy[tiab] OR ischaemic seizure[tiab] OR ischaemic seizures[tiab] OR ischemic attack[tiab] OR ischemic attacks[tiab] OR ischemic encephalopathies[tiab] OR ischemic encephalopathy[tiab] OR ischemic seizure[tiab] OR ischemic seizures[tiab] OR moya moya[tiab] OR moyamoya[tiab] OR neural ischaemia[tiab] OR neural ischemia[tiab] OR posterior fossa haemorrhag*[tiab] OR posterior fossa hemorrhag*[tiab] OR poststroke[tiab] OR poststrokes[tiab] OR stroke[tiab] OR strokes[tiab] OR thrombosis cerebri[tiab] OR ((brain[tiab] OR cerebral[tiab] OR cerebro-vascular[tiab] OR cerebrovascular[tiab] OR cranio-cerebral[tiab] OR craniocerebral[tiab] OR hemisphere[tiab] OR hemispheric[tiab] OR inter-hemispheric[tiab] OR interhemispheric[tiab] OR intra-cerebral[tiab] OR intra-cranial[tiab] OR intra-ventricular[tiab] OR intracerebral[tiab] OR intracranial[tiab] OR intraventricular[tiab] OR peri-ventricular[tiab] OR periventricular[tiab]) AND (accident[tiab] OR accidents[tiab] OR angiospas*[tiab] OR arrest[tiab] OR attack[tiab] OR attacks[tiab] OR bleed*[tiab] OR blood flow disorder[tiab] OR blood flow disorders[tiab] OR blood flow disturbance[tiab] OR blood flow disturbances[tiab] OR circulation disease[tiab] OR circulation diseases[tiab] OR circulation disorder[tiab] OR circulation disorders[tiab] OR circulation disturbance[tiab] OR circulation disturbances[tiab] OR circulatory disease[tiab] OR circulatory diseases[tiab] OR circulatory disorder[tiab] OR circulatory disorders[tiab] OR circulatory disturbance[tiab] OR circulatory disturbances[tiab] OR emboli*[tiab] OR embolus[tiab] OR failure[tiab] OR failures[tiab] OR haematoma*[tiab] OR haemorrhag*[tiab] OR hematoma*[tiab] OR hemorrhag*[tiab] OR infarct*[tiab] OR injur*[tiab] OR insufficien*[tiab] OR insult*[tiab] OR ischaemi*[tiab] OR ischemi*[tiab] OR micro-bleed*[tiab] OR micro-haemorrhag*[tiab] OR micro-hemorrhag*[tiab] OR microbleed*[tiab] OR microhaemorrhag*[tiab] OR microhemorrhag*[tiab] OR obstruct*[tiab] OR occlus*[tiab] OR spasm[tiab] OR spasms[tiab] OR thrombo*[tiab] OR vasospas*[tiab]))) AND ("Adolescent"[Mesh] OR "Child"[Mesh] OR "Infant"[Mesh] OR "Pediatrics"[Mesh] OR adolescen*[tiab] OR babies[tiab] OR baby[tiab] OR boy[tiab] OR boyhood[tiab] OR boys[tiab] OR child*[tiab] OR girl[tiab] OR girlhood[tiab] OR girls[tiab] OR infan*[tiab] OR juvenile[tiab] OR juveniles[tiab] OR kid[tiab] OR kids[tiab] OR minor[tiab] OR minors[tiab] OR neo-nat*[tiab] OR neonat*[tiab] OR new-born*[tiab] OR newborn*[tiab] OR paediatric*[tiab] OR pediatric*[tiab] OR pre-adolescen*[tiab] OR pre-school*[tiab] OR preadolescen*[tiab] OR preschool*[tiab] OR pubertal[tiab] OR puberty[tiab] OR pubescen*[tiab] OR school age*[tiab] OR school child*[tiab] OR schoolage*[tiab] OR schoolchild*[tiab] OR student[tiab] OR students[tiab] OR young[tiab] OR youngadult*[tiab] OR teen*[tiab] OR toddler[tiab] OR toddlers[tiab] OR under-age*[tiab] OR underage*[tiab] OR youth*[tiab]) |
| --- | --- |
| **Cumulative Index of Nursing and Allied Health Literature (CINAHL) Ultimate (EBSCOhost):**  **Results: 536**  **Limits/Filters: Children** | (MH "Anemia, Sickle Cell+" OR MH "Sickle Cell Trait" OR TI (drepanocyte OR drepanocytemia OR drepanocytes OR drepanocytic anaemia OR drepanocytic anemia OR drepanocytosis OR haemoglobin c OR haemoglobin d OR haemoglobin s OR "haemoglobin sc" OR haemoglobin sd OR haemoglobin ss OR Hb C OR Hb D OR Hb S OR "Hb SC" OR Hb SD OR Hb SS OR HbC OR HbD OR HbS OR HbSC OR HbSD OR HbSS OR hemoglobin c OR hemoglobin d OR hemoglobin s OR "hemoglobin sc" OR hemoglobin sd OR hemoglobin ss OR hemoglobinopathy c OR hemoglobinopathy d OR hemoglobinopathy s OR "hemoglobinopathy sc" OR hemoglobinopathy sd OR hemoglobinopathy ss OR meniscocyte OR meniscocytes OR meniscocytosis OR sickle anaemia OR sickle anemia OR sickle cell OR sickle cells OR sickle erythrocyte OR sickle erythrocytes OR sickled OR sickler OR sickling trait) OR AB (drepanocyte OR drepanocytemia OR drepanocytes OR drepanocytic anaemia OR drepanocytic anemia OR drepanocytosis OR haemoglobin c OR haemoglobin d OR haemoglobin s OR "haemoglobin sc" OR haemoglobin sd OR haemoglobin ss OR Hb C OR Hb D OR Hb S OR "Hb SC" OR Hb SD OR Hb SS OR HbC OR HbD OR HbS OR HbSC OR HbSD OR HbSS OR hemoglobin c OR hemoglobin d OR hemoglobin s OR "hemoglobin sc" OR hemoglobin sd OR hemoglobin ss OR hemoglobinopathy c OR hemoglobinopathy d OR hemoglobinopathy s OR "hemoglobinopathy sc" OR hemoglobinopathy sd OR hemoglobinopathy ss OR meniscocyte OR meniscocytes OR meniscocytosis OR sickle anaemia OR sickle anemia OR sickle cell OR sickle cells OR sickle erythrocyte OR sickle erythrocytes OR sickled OR sickler OR sickling trait)) AND (MH "Cerebral Ischemia+" OR MH "Intracranial Embolism and Thrombosis+" OR MH "Intracranial Hemorrhage+" OR MH "Moyamoya Disease" OR MH "Stroke+" OR MH "Stroke Patients" OR TI (acute cerebrovascular lesion OR acute cerebrovascular lesions OR acute focal cerebral vasculopath* OR apoplectic OR apoplex* OR brain event OR brain events OR cardio-vascular event OR cardio-vascular events OR cardiovascular event OR cardiovascular events OR corpus callosum hemorrhag* OR cortical infarct* OR CVA OR CVAs OR encephalorrhagia OR hematencephalon OR insultus cerebralis OR intracortical hemorrhag* OR ischaemic attack OR ischaemic attacks OR ischaemic encephalopathies OR ischaemic encephalopathy OR ischaemic seizure OR ischaemic seizures OR ischemic attack OR ischemic attacks OR ischemic encephalopathies OR ischemic encephalopathy OR ischemic seizure OR ischemic seizures OR moya moya OR moyamoya OR neural ischaemia OR neural ischemia OR posterior fossa haemorrhag* OR posterior fossa hemorrhag* OR poststroke OR poststrokes OR stroke OR strokes OR thrombosis cerebri) OR AB (acute cerebrovascular lesion OR acute cerebrovascular lesions OR acute focal cerebral vasculopath* OR apoplectic OR apoplex* OR brain event OR brain events OR cardio-vascular event OR cardio-vascular events OR cardiovascular event OR cardiovascular events OR corpus callosum hemorrhag* OR cortical infarct* OR CVA OR CVAs OR encephalorrhagia OR hematencephalon OR insultus cerebralis OR intracortical hemorrhag* OR ischaemic attack OR ischaemic attacks OR ischaemic encephalopathies OR ischaemic encephalopathy OR ischaemic seizure OR ischaemic seizures OR ischemic attack OR ischemic attacks OR ischemic encephalopathies OR ischemic encephalopathy OR ischemic seizure OR ischemic seizures OR moya moya OR moyamoya OR neural ischaemia OR neural ischemia OR posterior fossa haemorrhag* OR posterior fossa hemorrhag* OR poststroke OR poststrokes OR stroke OR strokes OR thrombosis cerebri) OR ((TI (brain OR cerebral OR cerebro-vascular OR cerebrovascular OR cranio-cerebral OR craniocerebral OR hemisphere OR hemispheric OR inter-hemispheric OR interhemispheric OR intra-cerebral OR intra-cranial OR intra-ventricular OR intracerebral OR intracranial OR intraventricular OR peri-ventricular OR periventricular) OR AB (brain OR cerebral OR cerebro-vascular OR cerebrovascular OR cranio-cerebral OR craniocerebral OR hemisphere OR hemispheric OR inter-hemispheric OR interhemispheric OR intra-cerebral OR intra-cranial OR intra-ventricular OR intracerebral OR intracranial OR intraventricular OR peri-ventricular OR periventricular)) AND (TI (accident OR accidents OR angiospas* OR arrest OR attack OR attacks OR bleed* OR blood flow disorder OR blood flow disorders OR blood flow disturbance OR blood flow disturbances OR circulation disease OR circulation diseases OR circulation disorder OR circulation disorders OR circulation disturbance OR circulation disturbances OR circulatory disease OR circulatory diseases OR circulatory disorder OR circulatory disorders OR circulatory disturbance OR circulatory disturbances OR emboli* OR embolus OR failure OR failures OR haematoma* OR haemorrhag* OR hematoma* OR hemorrhag* OR infarct* OR injur* OR insufficien* OR insult* OR ischaemi* OR ischemi* OR micro-bleed* OR micro-haemorrhag* OR micro-hemorrhag* OR microbleed* OR microhaemorrhag* OR microhemorrhag* OR obstruct* OR occlus* OR spasm OR spasms OR thrombo* OR vasospas*) OR AB (accident OR accidents OR angiospas* OR arrest OR attack OR attacks OR bleed* OR blood flow disorder OR blood flow disorders OR blood flow disturbance OR blood flow disturbances OR circulation disease OR circulation diseases OR circulation disorder OR circulation disorders OR circulation disturbance OR circulation disturbances OR circulatory disease OR circulatory diseases OR circulatory disorder OR circulatory disorders OR circulatory disturbance OR circulatory disturbances OR emboli* OR embolus OR failure OR failures OR haematoma* OR haemorrhag* OR hematoma* OR hemorrhag* OR infarct* OR injur* OR insufficien* OR insult* OR ischaemi* OR ischemi* OR micro-bleed* OR micro-haemorrhag* OR micro-hemorrhag* OR microbleed* OR microhaemorrhag* OR microhemorrhag* OR obstruct* OR occlus* OR spasm OR spasms OR thrombo* OR vasospas*)))) AND (MH "Adolescence+" OR MH "Child+" OR MH "Infant+" OR MH "Pediatrics+" OR TI (adolescen* OR babies OR baby OR boy OR boyhood OR boys OR child* OR girl OR girlhood OR girls OR infan* OR juvenile OR juveniles OR kid OR kids OR minor OR minors OR neo-nat* OR neonat* OR new-born* OR newborn* OR paediatric* OR pediatric* OR pre-adolescen* OR pre-school* OR preadolescen* OR preschool* OR pubertal OR puberty OR pubescen* OR school age* OR school child* OR schoolage* OR schoolchild* OR student OR students OR young OR youngadult* OR teen* OR toddler OR toddlers OR under-age* OR underage* OR youth*) OR AB (adolescen* OR babies OR baby OR boy OR boyhood OR boys OR child* OR girl OR girlhood OR girls OR infan* OR juvenile OR juveniles OR kid OR kids OR minor OR minors OR neo-nat* OR neonat* OR new-born* OR newborn* OR paediatric* OR pediatric* OR pre-adolescen* OR pre-school* OR preadolescen* OR preschool* OR pubertal OR puberty OR pubescen* OR school age* OR school child* OR schoolage* OR schoolchild* OR student OR students OR young OR youngadult* OR teen* OR toddler OR toddlers OR under-age* OR underage* OR youth*)) |
| **Embase (Elsevier):**  **Results: 4,027**  **Limits/Filters: Children** | ('sickle cell'/exp OR 'sickle cell anemia'/exp OR drepanocyte:ab,kw,ti OR drepanocytemia:ab,kw,ti OR drepanocytes:ab,kw,ti OR 'drepanocytic anaemia':ab,kw,ti OR 'drepanocytic anemia':ab,kw,ti OR drepanocytosis:ab,kw,ti OR 'haemoglobin c':ab,kw,ti OR 'haemoglobin d':ab,kw,ti OR 'haemoglobin s':ab,kw,ti OR 'haemoglobin sc':ab,kw,ti OR 'haemoglobin sd':ab,kw,ti OR 'haemoglobin ss':ab,kw,ti OR 'hb c':ab,kw,ti OR 'hb d':ab,kw,ti OR 'hb s':ab,kw,ti OR 'hb sc':ab,kw,ti OR 'hb sd':ab,kw,ti OR 'hb ss':ab,kw,ti OR hbc:ab,kw,ti OR hbd:ab,kw,ti OR hbs:ab,kw,ti OR hbsc:ab,kw,ti OR hbsd:ab,kw,ti OR hbss:ab,kw,ti OR 'hemoglobin c':ab,kw,ti OR 'hemoglobin d':ab,kw,ti OR 'hemoglobin s':ab,kw,ti OR 'hemoglobin sc':ab,kw,ti OR 'hemoglobin sd':ab,kw,ti OR 'hemoglobin ss':ab,kw,ti OR 'hemoglobinopathy c':ab,kw,ti OR 'hemoglobinopathy d':ab,kw,ti OR 'hemoglobinopathy s':ab,kw,ti OR 'hemoglobinopathy sc':ab,kw,ti OR 'hemoglobinopathy sd':ab,kw,ti OR 'hemoglobinopathy ss':ab,kw,ti OR meniscocyte:ab,kw,ti OR meniscocytes:ab,kw,ti OR meniscocytosis:ab,kw,ti OR 'sickle anaemia':ab,kw,ti OR 'sickle anemia':ab,kw,ti OR 'sickle cell':ab,kw,ti OR 'sickle cells':ab,kw,ti OR 'sickle erythrocyte':ab,kw,ti OR 'sickle erythrocytes':ab,kw,ti OR sickled:ab,kw,ti OR sickler:ab,kw,ti OR 'sickling trait':ab,kw,ti) AND ('brain embolism'/exp OR 'brain hematoma'/exp OR 'brain hemorrhage'/exp OR 'brain infarction'/exp OR 'brain ischemia'/exp OR 'brain vasospasm'/exp OR 'cerebral thrombosis'/exp OR 'cerebrovascular accident'/exp OR 'moyamoya disease'/exp OR 'occlusive cerebrovascular disease'/exp OR 'stroke patient'/exp OR 'acute cerebrovascular lesion':ab,kw,ti OR 'acute cerebrovascular lesions':ab,kw,ti OR 'acute focal cerebral vasculopath*':ab,kw,ti OR apoplectic:ab,kw,ti OR apoplex*:ab,kw,ti OR 'brain event':ab,kw,ti OR 'brain events':ab,kw,ti OR 'cardio-vascular event':ab,kw,ti OR 'cardio-vascular events':ab,kw,ti OR 'cardiovascular event':ab,kw,ti OR 'cardiovascular events':ab,kw,ti OR 'corpus callosum hemorrhag*':ab,kw,ti OR 'cortical infarct*':ab,kw,ti OR cva:ab,kw,ti OR cvas:ab,kw,ti OR encephalorrhagia:ab,kw,ti OR hematencephalon:ab,kw,ti OR 'insultus cerebralis':ab,kw,ti OR 'intracortical hemorrhag*':ab,kw,ti OR 'ischaemic attack':ab,kw,ti OR 'ischaemic attacks':ab,kw,ti OR 'ischaemic encephalopathies':ab,kw,ti OR 'ischaemic encephalopathy':ab,kw,ti OR 'ischaemic seizure':ab,kw,ti OR 'ischaemic seizures':ab,kw,ti OR 'ischemic attack':ab,kw,ti OR 'ischemic attacks':ab,kw,ti OR 'ischemic encephalopathies':ab,kw,ti OR 'ischemic encephalopathy':ab,kw,ti OR 'ischemic seizure':ab,kw,ti OR 'ischemic seizures':ab,kw,ti OR 'moya moya':ab,kw,ti OR moyamoya:ab,kw,ti OR 'neural ischaemia':ab,kw,ti OR 'neural ischemia':ab,kw,ti OR 'posterior fossa haemorrhag*':ab,kw,ti OR 'posterior fossa hemorrhag*':ab,kw,ti OR poststroke:ab,kw,ti OR poststrokes:ab,kw,ti OR stroke:ab,kw,ti OR strokes:ab,kw,ti OR 'thrombosis cerebri':ab,kw,ti OR ((brain:ab,kw,ti OR cerebral:ab,kw,ti OR 'cerebro vascular':ab,kw,ti OR cerebrovascular:ab,kw,ti OR 'cranio cerebral':ab,kw,ti OR craniocerebral:ab,kw,ti OR hemisphere:ab,kw,ti OR hemispheric:ab,kw,ti OR 'inter hemispheric':ab,kw,ti OR interhemispheric:ab,kw,ti OR 'intra cerebral':ab,kw,ti OR 'intra cranial':ab,kw,ti OR 'intra ventricular':ab,kw,ti OR intracerebral:ab,kw,ti OR intracranial:ab,kw,ti OR intraventricular:ab,kw,ti OR 'peri ventricular':ab,kw,ti OR periventricular:ab,kw,ti) AND (accident:ab,kw,ti OR accidents:ab,kw,ti OR angiospas*:ab,kw,ti OR arrest:ab,kw,ti OR attack:ab,kw,ti OR attacks:ab,kw,ti OR bleed*:ab,kw,ti OR 'blood flow disorder':ab,kw,ti OR 'blood flow disorders':ab,kw,ti OR 'blood flow disturbance':ab,kw,ti OR 'blood flow disturbances':ab,kw,ti OR 'circulation disease':ab,kw,ti OR 'circulation diseases':ab,kw,ti OR 'circulation disorder':ab,kw,ti OR 'circulation disorders':ab,kw,ti OR 'circulation disturbance':ab,kw,ti OR 'circulation disturbances':ab,kw,ti OR 'circulatory disease':ab,kw,ti OR 'circulatory diseases':ab,kw,ti OR 'circulatory disorder':ab,kw,ti OR 'circulatory disorders':ab,kw,ti OR 'circulatory disturbance':ab,kw,ti OR 'circulatory disturbances':ab,kw,ti OR emboli*:ab,kw,ti OR embolus:ab,kw,ti OR failure:ab,kw,ti OR failures:ab,kw,ti OR haematoma*:ab,kw,ti OR haemorrhag*:ab,kw,ti OR hematoma*:ab,kw,ti OR hemorrhag*:ab,kw,ti OR infarct*:ab,kw,ti OR injur*:ab,kw,ti OR insufficien*:ab,kw,ti OR insult*:ab,kw,ti OR ischaemi*:ab,kw,ti OR ischemi*:ab,kw,ti OR 'micro bleed*':ab,kw,ti OR 'micro haemorrhag*':ab,kw,ti OR 'micro hemorrhag*':ab,kw,ti OR microbleed*:ab,kw,ti OR microhaemorrhag*:ab,kw,ti OR microhemorrhag*:ab,kw,ti OR obstruct*:ab,kw,ti OR occlus*:ab,kw,ti OR spasm:ab,kw,ti OR spasms:ab,kw,ti OR thrombo*:ab,kw,ti OR vasospas*:ab,kw,ti))) AND ('adolescent'/exp OR 'child'/exp OR 'pediatrics'/exp OR adolescen*:ab,kw,ti OR babies:ab,kw,ti OR baby:ab,kw,ti OR boy:ab,kw,ti OR boyhood:ab,kw,ti OR boys:ab,kw,ti OR child*:ab,kw,ti OR girl:ab,kw,ti OR girlhood:ab,kw,ti OR girls:ab,kw,ti OR infan*:ab,kw,ti OR juvenile:ab,kw,ti OR juveniles:ab,kw,ti OR kid:ab,kw,ti OR kids:ab,kw,ti OR minor:ab,kw,ti OR minors:ab,kw,ti OR 'neo nat*':ab,kw,ti OR neonat*:ab,kw,ti OR 'new born*':ab,kw,ti OR newborn*:ab,kw,ti OR paediatric*:ab,kw,ti OR pediatric*:ab,kw,ti OR 'pre adolescen*':ab,kw,ti OR 'pre school*':ab,kw,ti OR preadolescen*:ab,kw,ti OR preschool*:ab,kw,ti OR pubertal:ab,kw,ti OR puberty:ab,kw,ti OR pubescen*:ab,kw,ti OR 'school age*':ab,kw,ti OR 'school child*':ab,kw,ti OR schoolage*:ab,kw,ti OR schoolchild*:ab,kw,ti OR student:ab,kw,ti OR students:ab,kw,ti OR young:ab,kw,ti OR youngadult*:ab,kw,ti OR teen*:ab,kw,ti OR toddler:ab,kw,ti OR toddlers:ab,kw,ti OR 'under age*':ab,kw,ti OR underage*:ab,kw,ti OR youth*:ab,kw,ti) |
| **Web of Science, Core Collection (Clarivate):**  **Results: 2,372**  **Limits/Filters: Children** | TS=(drepanocyte OR drepanocytemia OR drepanocytes OR "drepanocytic anaemia" OR "drepanocytic anemia" OR drepanocytosis OR "haemoglobin c" OR "haemoglobin d" OR "haemoglobin s" OR "haemoglobin sc" OR "haemoglobin sd" OR "haemoglobin ss" OR "Hb C" OR "Hb D" OR "Hb S" OR "Hb SC" OR "Hb SD" OR "Hb SS" OR HbC OR HbD OR HbS OR HbSC OR HbSD OR HbSS OR "hemoglobin c" OR "hemoglobin d" OR "hemoglobin s" OR "hemoglobin sc" OR "hemoglobin sd" OR "hemoglobin ss" OR "hemoglobinopathy c" OR "hemoglobinopathy d" OR "hemoglobinopathy s" OR "hemoglobinopathy sc" OR "hemoglobinopathy sd" OR "hemoglobinopathy ss" OR meniscocyte OR meniscocytes OR meniscocytosis OR "sickle anaemia" OR "sickle anemia" OR "sickle cell" OR "sickle cells" OR "sickle erythrocyte" OR "sickle erythrocytes" OR sickled OR sickler OR "sickling trait") AND TS=("acute cerebrovascular lesion" OR "acute cerebrovascular lesions" OR "acute focal cerebral vasculopath*" OR apoplectic OR apoplex* OR "brain event" OR "brain events" OR "cardio-vascular event" OR "cardio-vascular events" OR "cardiovascular event" OR "cardiovascular events" OR "corpus callosum hemorrhag*" OR "cortical infarct*" OR CVA OR CVAs OR encephalorrhagia OR hematencephalon OR "insultus cerebralis" OR "intracortical hemorrhag*" OR "ischaemic attack" OR "ischaemic attacks" OR "ischaemic encephalopathies" OR "ischaemic encephalopathy" OR "ischaemic seizure" OR "ischaemic seizures" OR "ischemic attack" OR "ischemic attacks" OR "ischemic encephalopathies" OR "ischemic encephalopathy" OR "ischemic seizure" OR "ischemic seizures" OR "moya moya" OR moyamoya OR "neural ischaemia" OR "neural ischemia" OR "posterior fossa haemorrhag*" OR "posterior fossa hemorrhag*" OR poststroke OR poststrokes OR stroke OR strokes OR "thrombosis cerebri" OR ((brain OR cerebral OR cerebro-vascular OR cerebrovascular OR cranio-cerebral OR craniocerebral OR hemisphere OR hemispheric OR inter-hemispheric OR interhemispheric OR intra-cerebral OR intra-cranial OR intra-ventricular OR intracerebral OR intracranial OR intraventricular OR peri-ventricular OR periventricular) AND (accident OR accidents OR angiospas* OR arrest OR attack OR attacks OR bleed* OR "blood flow disorder" OR "blood flow disorders" OR "blood flow disturbance" OR "blood flow disturbances" OR "circulation disease" OR "circulation diseases" OR "circulation disorder" OR "circulation disorders" OR "circulation disturbance" OR "circulation disturbances" OR "circulatory disease" OR "circulatory diseases" OR "circulatory disorder" OR "circulatory disorders" OR "circulatory disturbance" OR "circulatory disturbances" OR emboli* OR embolus OR failure OR failures OR haematoma* OR haemorrhag* OR hematoma* OR hemorrhag* OR infarct* OR injur* OR insufficien* OR insult* OR ischaemi* OR ischemi* OR micro-bleed* OR micro-haemorrhag* OR micro-hemorrhag* OR microbleed* OR microhaemorrhag* OR microhemorrhag* OR obstruct* OR occlus* OR spasm OR spasms OR thrombo* OR vasospas*))) AND TS=(adolescen* OR babies OR baby OR boy OR boyhood OR boys OR child* OR girl OR girlhood OR girls OR infan* OR juvenile OR juveniles OR kid OR kids OR minor OR minors OR neo-nat* OR neonat* OR new-born* OR newborn* OR paediatric* OR pediatric* OR pre-adolescen* OR pre-school* OR preadolescen* OR preschool* OR pubertal OR puberty OR pubescen* OR "school age*" OR "school child*" OR schoolage* OR schoolchild* OR student OR students OR young OR youngadult* OR teen* OR toddler OR toddlers OR under-age* OR underage* OR youth*) |
| **Cochrane CENTRAL Register of Controlled Trials (Wiley):**  **Results: 238**  **Limits/Filters: Children** | ([mh "Anemia, Sickle Cell"] OR (drepanocyte OR drepanocytemia OR drepanocytes OR "drepanocytic anaemia" OR "drepanocytic anemia" OR drepanocytosis OR "haemoglobin c" OR "haemoglobin d" OR "haemoglobin s" OR "haemoglobin sc" OR "haemoglobin sd" OR "haemoglobin ss" OR "Hb C" OR "Hb D" OR "Hb S" OR "Hb SC" OR "Hb SD" OR "Hb SS" OR HbC OR HbD OR HbS OR HbSC OR HbSD OR HbSS OR "hemoglobin c" OR "hemoglobin d" OR "hemoglobin s" OR "hemoglobin sc" OR "hemoglobin sd" OR "hemoglobin ss" OR "hemoglobinopathy c" OR "hemoglobinopathy d" OR "hemoglobinopathy s" OR "hemoglobinopathy sc" OR "hemoglobinopathy sd" OR "hemoglobinopathy ss" OR meniscocyte OR meniscocytes OR meniscocytosis OR "sickle anaemia" OR "sickle anemia" OR "sickle cell" OR "sickle cells" OR "sickle erythrocyte" OR "sickle erythrocytes" OR sickled OR sickler OR "sickling trait"):ti,ab,kw) AND ([mh "Brain Infarction"] OR [mh "Brain Ischemia"] OR [mh "Intracranial Embolism and Thrombosis"] OR [mh "Intracranial Hemorrhages"] OR [mh "Moyamoya Disease"] OR [mh "Stroke"] OR [mh "Vasospasm, Intracranial"] OR ("acute cerebrovascular lesion" OR "acute cerebrovascular lesions" OR (acute NEXT focal NEXT cerebral NEXT vasculopath*) OR apoplectic OR apoplex* OR "brain event" OR "brain events" OR "cardio-vascular event" OR "cardio-vascular events" OR "cardiovascular event" OR "cardiovascular events" OR (corpus NEXT callosum NEXT hemorrhag*) OR (cortical NEXT infarct*) OR CVA OR CVAs OR encephalorrhagia OR hematencephalon OR "insultus cerebralis" OR (intracortical NEXT hemorrhag*) OR "ischaemic attack" OR "ischaemic attacks" OR "ischaemic encephalopathies" OR "ischaemic encephalopathy" OR "ischaemic seizure" OR "ischaemic seizures" OR "ischemic attack" OR "ischemic attacks" OR "ischemic encephalopathies" OR "ischemic encephalopathy" OR "ischemic seizure" OR "ischemic seizures" OR "moya moya" OR moyamoya OR "neural ischaemia" OR "neural ischemia" OR (posterior NEXT fossa NEXT haemorrhag*) OR (posterior NEXT fossa NEXT hemorrhag*) OR poststroke OR poststrokes OR stroke OR strokes OR "thrombosis cerebri"):ti,ab,kw OR ((brain OR cerebral OR cerebro-vascular OR cerebrovascular OR cranio-cerebral OR craniocerebral OR hemisphere OR hemispheric OR inter-hemispheric OR interhemispheric OR intra-cerebral OR intra-cranial OR intra-ventricular OR intracerebral OR intracranial OR intraventricular OR peri-ventricular OR periventricular):ti,ab,kw AND (accident OR accidents OR angiospas* OR arrest OR attack OR attacks OR bleed* OR "blood flow disorder" OR "blood flow disorders" OR "blood flow disturbance" OR "blood flow disturbances" OR "circulation disease" OR "circulation diseases" OR "circulation disorder" OR "circulation disorders" OR "circulation disturbance" OR "circulation disturbances" OR "circulatory disease" OR "circulatory diseases" OR "circulatory disorder" OR "circulatory disorders" OR "circulatory disturbance" OR "circulatory disturbances" OR emboli* OR embolus OR failure OR failures OR haematoma* OR haemorrhag* OR hematoma* OR hemorrhag* OR infarct* OR injur* OR insufficien* OR insult* OR ischaemi* OR ischemi* OR micro-bleed* OR micro-haemorrhag* OR micro-hemorrhag* OR microbleed* OR microhaemorrhag* OR microhemorrhag* OR obstruct* OR occlus* OR spasm OR spasms OR thrombo* OR vasospas*):ti,ab,kw)) AND ([mh "Adolescent"] OR [mh "Child"] OR [mh "Infant"] OR [mh "Pediatrics"] OR (adolescen* OR babies OR baby OR boy OR boyhood OR boys OR child* OR girl OR girlhood OR girls OR infan* OR juvenile OR juveniles OR kid OR kids OR minor OR minors OR neo-nat* OR neonat* OR new-born* OR newborn* OR paediatric* OR pediatric* OR pre-adolescen* OR pre-school* OR preadolescen* OR preschool* OR pubertal OR puberty OR pubescen* OR (school NEXT age*) OR (school NEXT child*) OR schoolage* OR schoolchild* OR student OR students OR young OR youngadult* OR teen* OR toddler OR toddlers OR under-age* OR underage* OR youth*):ti,ab,kw) |
| **Cochrane Database of Systematic Reviews (Wiley):**  **Results: 12**  **Limits/Filters: Children** | ([mh "Anemia, Sickle Cell"] OR (drepanocyte OR drepanocytemia OR drepanocytes OR "drepanocytic anaemia" OR "drepanocytic anemia" OR drepanocytosis OR "haemoglobin c" OR "haemoglobin d" OR "haemoglobin s" OR "haemoglobin sc" OR "haemoglobin sd" OR "haemoglobin ss" OR "Hb C" OR "Hb D" OR "Hb S" OR "Hb SC" OR "Hb SD" OR "Hb SS" OR HbC OR HbD OR HbS OR HbSC OR HbSD OR HbSS OR "hemoglobin c" OR "hemoglobin d" OR "hemoglobin s" OR "hemoglobin sc" OR "hemoglobin sd" OR "hemoglobin ss" OR "hemoglobinopathy c" OR "hemoglobinopathy d" OR "hemoglobinopathy s" OR "hemoglobinopathy sc" OR "hemoglobinopathy sd" OR "hemoglobinopathy ss" OR meniscocyte OR meniscocytes OR meniscocytosis OR "sickle anaemia" OR "sickle anemia" OR "sickle cell" OR "sickle cells" OR "sickle erythrocyte" OR "sickle erythrocytes" OR sickled OR sickler OR "sickling trait"):ti,ab,kw) AND ([mh "Brain Infarction"] OR [mh "Brain Ischemia"] OR [mh "Intracranial Embolism and Thrombosis"] OR [mh "Intracranial Hemorrhages"] OR [mh "Moyamoya Disease"] OR [mh "Stroke"] OR [mh "Vasospasm, Intracranial"] OR ("acute cerebrovascular lesion" OR "acute cerebrovascular lesions" OR (acute NEXT focal NEXT cerebral NEXT vasculopath*) OR apoplectic OR apoplex* OR "brain event" OR "brain events" OR "cardio-vascular event" OR "cardio-vascular events" OR "cardiovascular event" OR "cardiovascular events" OR (corpus NEXT callosum NEXT hemorrhag*) OR (cortical NEXT infarct*) OR CVA OR CVAs OR encephalorrhagia OR hematencephalon OR "insultus cerebralis" OR (intracortical NEXT hemorrhag*) OR "ischaemic attack" OR "ischaemic attacks" OR "ischaemic encephalopathies" OR "ischaemic encephalopathy" OR "ischaemic seizure" OR "ischaemic seizures" OR "ischemic attack" OR "ischemic attacks" OR "ischemic encephalopathies" OR "ischemic encephalopathy" OR "ischemic seizure" OR "ischemic seizures" OR "moya moya" OR moyamoya OR "neural ischaemia" OR "neural ischemia" OR (posterior NEXT fossa NEXT haemorrhag*) OR (posterior NEXT fossa NEXT hemorrhag*) OR poststroke OR poststrokes OR stroke OR strokes OR "thrombosis cerebri"):ti,ab,kw OR ((brain OR cerebral OR cerebro-vascular OR cerebrovascular OR cranio-cerebral OR craniocerebral OR hemisphere OR hemispheric OR inter-hemispheric OR interhemispheric OR intra-cerebral OR intra-cranial OR intra-ventricular OR intracerebral OR intracranial OR intraventricular OR peri-ventricular OR periventricular):ti,ab,kw AND (accident OR accidents OR angiospas* OR arrest OR attack OR attacks OR bleed* OR "blood flow disorder" OR "blood flow disorders" OR "blood flow disturbance" OR "blood flow disturbances" OR "circulation disease" OR "circulation diseases" OR "circulation disorder" OR "circulation disorders" OR "circulation disturbance" OR "circulation disturbances" OR "circulatory disease" OR "circulatory diseases" OR "circulatory disorder" OR "circulatory disorders" OR "circulatory disturbance" OR "circulatory disturbances" OR emboli* OR embolus OR failure OR failures OR haematoma* OR haemorrhag* OR hematoma* OR hemorrhag* OR infarct* OR injur* OR insufficien* OR insult* OR ischaemi* OR ischemi* OR micro-bleed* OR micro-haemorrhag* OR micro-hemorrhag* OR microbleed* OR microhaemorrhag* OR microhemorrhag* OR obstruct* OR occlus* OR spasm OR spasms OR thrombo* OR vasospas*):ti,ab,kw)) AND ([mh "Adolescent"] OR [mh "Child"] OR [mh "Infant"] OR [mh "Pediatrics"] OR (adolescen* OR babies OR baby OR boy OR boyhood OR boys OR child* OR girl OR girlhood OR girls OR infan* OR juvenile OR juveniles OR kid OR kids OR minor OR minors OR neo-nat* OR neonat* OR new-born* OR newborn* OR paediatric* OR pediatric* OR pre-adolescen* OR pre-school* OR preadolescen* OR preschool* OR pubertal OR puberty OR pubescen* OR (school NEXT age*) OR (school NEXT child*) OR schoolage* OR schoolchild* OR student OR students OR young OR youngadult* OR teen* OR toddler OR toddlers OR under-age* OR underage* OR youth*):ti,ab,kw) |
| **WHO Global Index Medicus:**  **Results: 138**  **Limits/Filters: None** | (drepanocyte OR drepanocytemia OR drepanocytes OR "drepanocytic anaemia" OR "drepanocytic anemia" OR drepanocytosis OR "haemoglobin c" OR "haemoglobin d" OR "haemoglobin s" OR "haemoglobin sc" OR "haemoglobin sd" OR "haemoglobin ss" OR "Hb C" OR "Hb D" OR "Hb S" OR "Hb SC" OR "Hb SD" OR "Hb SS" OR HbC OR HbD OR HbS OR HbSC OR HbSD OR HbSS OR "hemoglobin c" OR "hemoglobin d" OR "hemoglobin s" OR "hemoglobin sc" OR "hemoglobin sd" OR "hemoglobin ss" OR "hemoglobinopathy c" OR "hemoglobinopathy d" OR "hemoglobinopathy s" OR "hemoglobinopathy sc" OR "hemoglobinopathy sd" OR "hemoglobinopathy ss" OR meniscocyte OR meniscocytes OR meniscocytosis OR "sickle anaemia" OR "sickle anemia" OR "sickle cell" OR "sickle cells" OR "sickle erythrocyte" OR "sickle erythrocytes" OR sickled OR sickler OR "sickling trait") AND ("cerebral infarct" OR "cerebral infarction" OR "cerebral infarctions" OR "cerebral infarcts" OR "cerebral ischaemia" OR "cerebral ischaemias" OR "cerebral ischemia" OR "cerebral ischemias" OR "cerebro-vascular accident" OR "cerebro-vascular accidents" OR "cerebro-vascular disease" OR "cerebro-vascular diseases" OR "cerebro-vascular event" OR "cerebro-vascular events" OR "cerebrovascular accident" OR "cerebrovascular accidents" OR "cerebrovascular disease" OR "cerebrovascular diseases" OR "cerebrovascular event" OR "cerebrovascular events" OR "intracranial embolism" OR "intracranial embolisms" OR "moya moya" OR moyamoya OR stroke OR strokes) |
